# Evolution of Therapy for ANCA-Associated Vasculitis with Kidney Involvement

**DOI:** 10.34067/KID.0000000000000289

**Published:** 2023-11-06

**Authors:** Arun Rajasekaran, Dana V. Rizk

**Affiliations:** Division of Nephrology, Department of Medicine, Heersink School of Medicine, University of Alabama at Birmingham, Birmingham, Alabama

**Keywords:** ANCA, ANCA-associated vasculitis, autoantibody, treatment

## Abstract

ANCA-associated vasculitis (AAV) belongs to a group of small vessel systemic vasculitides characterized by granulomatous and neutrophilic inflammation of various tissues. Patients often have circulating autoantibodies targeting neutrophilic antigens. Although AAV was once associated with severe end-organ damage and extremely high mortality rates, the use of glucocorticoids and cyclophosphamide led to a paradigm change in its treatment. Over the past 20 years, significant progress in understanding the immunopathogenesis of AAV has enabled development of targeted immunotherapies, providing a much better prognosis for patients. This review describes the evolution of treatment of AAV, particularly for patients with kidney involvement.

## Introduction

Systemic vasculitis (eventually termed polyarteritis nodosa) was first reported in 1866 when Kussmaul and Maier described nodular thickening of countless arteries in the autopsy of a patient.^1,2^ In 1931, Klinger reported a case of systemic vasculitis involving the respiratory tract and kidneys that would later be called Wegener granulomatosis (WG) after the 1936 description of patients with prominent nasal lesions, GN, granulomatous inflammation, and vasculitis.^3^ In 1951, Churg and Strauss described 13 patients with microscopic polyarteritis and eosinophilic vasculitis (now termed microscopic polyangiitis [MPA]) manifesting as focal segmental necrotizing GN as a separate entity from polyarteritis nodosa, a syndrome that bore their names for years.^4^

The etiology of vasculitic diseases remained unknown until 1982 when Davies discovered a factor that stained the cytoplasm of neutrophil leukocytes by indirect immunofluorescence in sera from eight patients with necrotizing crescentic GN.^5^ In 1988, Falk and Jennette described two immunofluorescence patterns of ANCA: perinuclear, corresponding to myeloperoxidase reactivity on ELISA, and cytoplasmic, with no reactivity to myeloperoxidase.^6^ Shortly thereafter, a novel neutrophil serine proteinase was identified as the antigen responsible for the cytoplasmic pattern.^7^ These discoveries led to development of nomenclature and definitions of systemic vasculitides at the International Chapel Hill Consensus Conferences in 1994^8^ and 2012,^9^ refining the terminology of small vessel vasculitides as ANCA-associated vasculitis (AAV) and immune complex small vessel vasculitides. The major clinicopathological variants of AAV include MPA, granulomatosis with polyangiitis (GPA, formerly WG), and eosinophilic GPA (formerly Churg-Strauss syndrome).^9^ In addition, vasculitides are classified on the basis of extent and severity at presentation—localized, early systemic, generalized, severe, and refractory (Table 1).^9,10^ A pauci-immune GN defines AAV as generalized or severe.^10^

**Table 1:** Classification of vasculitis according to extent and severity at presentation

Disease Subgroup	Definition	ANCA Serology
Localized	Vasculitis confined to one organ system, no systemic disturbance	Often negative
Early systemic	Vasculitis in at least one organ system with systemic features without threatened vital organ function	Usually positive
Generalized	Vasculitis in at least one organ system with systemic features and threatened vital organ function	Usually positive
Severe	Vasculitis in at least one organ system with systemic features and vital organ function failure (SCr ≥500 *μ*mol/L [≥5.7 mg/dl] if kidney involvement)	Usually positive
Refractory	Failure of standard induction regimen	Positive or negative

SCr, serum creatinine.

The pathogenesis of AAV is complex, but substantial experimental and clinical evidence supports the pathogenic role of ANCA.^11,12^ Neutrophils can be primed by cytokines, leading to translocation of small amounts of ANCA autoantigens to their cell surface. Primed neutrophils adhere to vascular endothelial surfaces. Circulating ANCA interact with their target antigens, inducing neutrophil activation and degranulation on surfaces of small vessel endothelial cells. This process causes necrotizing small vessel injury and triggers a cascade of events amplified by release of inflammatory mediators, including complement-derived neutrophil and monocyte chemotactic factors, such as C5a, and factors activating the alternative complement pathway.^13^

**Figure 1:**
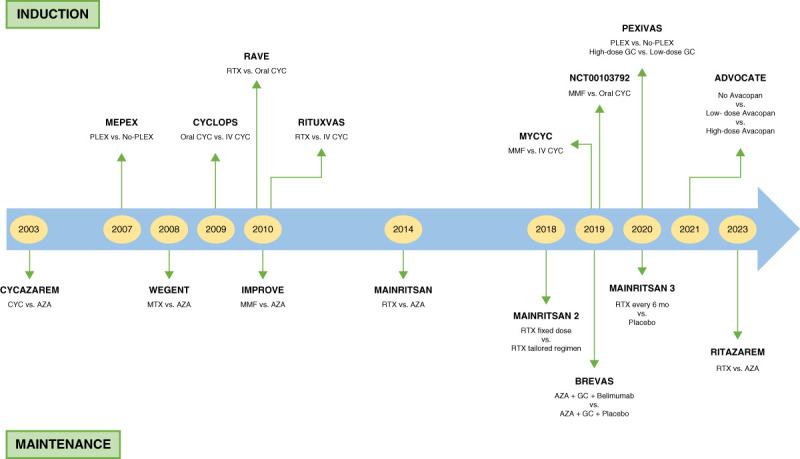
**Landmark phase III clinical trials for treatment of patients with AAV with kidney involvement.** The important phase III clinical trials for testing new therapies for induction of remission (above timeline) and maintenance of remission (below timeline) for patients with AAV with kidney involvement over the past two decades are shown. Timeline provides the year of publication of the reports of the results of the clinical trials. Details about the trials, including inclusion/exclusion criteria, intervention agents and dosing, primary outcomes, and major highlights, are provided in Table 2 (trials for induction of remission) and Table 3 (trials for maintenance of remission). AAV, ANCA-associated vasculitis; AZA, azathioprine; CYC, cyclophosphamide; GC, glucocorticoids; IV, intravenous; MMF, mycophenolate mofetil; MTX, methotrexate; NCT, national clinical trial identifier; PLEX, plasma exchange; RTX, rituximab.

Before treatment with immunosuppressive regimens, mortality of patients with severe AAV approached 80% within 1 year of diagnosis.^14^ Therapeutic advances improved prognosis; currently, the estimated 5-year survival is 74%–91% and 45%–76% for GPA and MPA, respectively.^15^ Because of the high risk of relapse, management of AAV consists of remission induction, maintenance, and relapse therapies.^12,16^ In this review, we describe the evolution of AAV treatment strategies, focusing on kidney involvement (Figure 1).

## Kidneys in AAV

The frequency and severity of kidney involvement in AAV varies according to clinical phenotype and ANCA serology. Kidney injury is classified as part of generalized vasculitic involvement when serum creatinine at presentation is <5.7 mg/dl (<500 *µ*mol/L) or severe when ≥5.7 mg/dl (≥500 *µ*mol/L).^10^ Up to 20%–25% of patients progress to kidney failure within a few years after diagnosis.^17^ Although cardiovascular disease and infections contribute to premature death,^18,19^ initial presentation and severity of kidney dysfunction are important predictors of mortality.^20–22^ In a study of 484 patients with AAV, the all-cause mortality rate was 38.4 per 1000 person-years. Compared with the general population, the standardized mortality ratio (SMR) for all-cause mortality was 2.3 (95% confidence interval, 1.9 to 2.8), and the leading cause of death was infections (SMR=13.9), followed by kidney disease–related mortality (SMR=4.3; 95% confidence interval, 1.6 to 11.3).^18^ Stabilizing kidney function is a paramount therapeutic goal.^23,24^

## Glucocorticoids—A Double-Edged Sword for AAV Induction Therapy

A 1958 retrospective description of 56 patients with WG (now GPA) documented an average survival of 5 months, with kidney and respiratory failure the leading causes of death.^25^ A literature review in 1967 described improved survival in 26 patients with GPA who received glucocorticoids (GCs) compared with no treatment (12.5 versus 5 months).^25,26^ GCs reduce inflammation and improve symptoms early in the course of AAV but confer little long-term benefit for lung and kidney involvement.^12^ Furthermore, serious adverse effects (SAEs) limit high-dose long-term therapy.^27^ Patients with AAV-related GN are usually treated with a tapering schedule of oral GCs.^28^ Most patients with AAV complicated by alveolar hemorrhage or rapidly progressive GN (RPGN) receive 2–3 pulses of intravenous (IV) methylprednisolone (MP), followed by a taper of oral GCs.^16^

## Introduction of Alkylating Agents

The first published case of GPA treatment with cytotoxic chemotherapy in 1954 described a 38-year-old male patient with predominantly pulmonary symptoms who markedly improved after IV nitrogen mustard and high-dose GCs.^29^ In a 1967 case report, a patient with GPA with severe lung and kidney injury attained complete remission after 1 year on prednisone and IV nitrogen mustard. The key observation was that high-dose GCs, although valuable early in AAV, were insufficient, and alkylating agents could potentially induce disease remission.^26^ The first report of cyclophosphamide (CYC) as single-drug therapy for GPA appeared in 1971.^30^ High-dose IV therapy preceded oral dosing for one patient; the other three received only oral therapy. Two patients were off immunosuppressants after 20 months without relapse, and the other two were stable without flare on low-dose treatment for 3.5 years.^30^

In a 1971 landmark publication, Fauci *et al.* at the National Institutes of Health (NIH) described nine patients with GPA who responded well to oral CYC alone (100–125 mg/d) for 1–39 months, therapy that induced humoral and cellular immunosuppression.^31^ In 1973, Fauci *et al.* described another 18 patients with systemic GPA, 15 with kidney involvement. Fourteen patients received oral CYC 1–2 mg/kg per day (after initial IV treatment in fulminant cases) and a GC taper in patients with severe inflammation. Among 12 patients with kidney involvement, one stopped dialysis, and ten attained complete remission (normal kidney function without microscopic hematuria) or partial remission (kidney function worse than baseline with absence of microscopic hematuria); one died from coronary vasculitis. Repeat kidney biopsies in a subset of patients 4 months to 5 years after treatment initiation documented significant histologic improvement in disease activity. Aware of potential serious toxicity related to cumulative CYC exposure, the authors recommended discontinuing cytotoxic treatment on remission.^32^

A subsequent NIH report of 85 patients with GPA treated with oral CYC and GC and followed for 21 years showed complete remission in 93% of the cohort. Oral CYC was given at 2 mg/kg per day for at least 1 year after the patient achieved complete remission. Subsequently, the dose was tapered and maintained at a level below which the disease flared. The mean treatment duration was 46.6 (±3.7) months. This study further documented that long-term remissions could be induced and maintained in GPA. Adverse effects included mild leukopenia and hemorrhagic cystitis; one patient developed diffuse histiocytic lymphoma.^33^

The NIH team published their collective experience with 180 patients with GPA with multiorgan AAV between 1967 and 1991.^34^ Most received standard therapy with oral CYC and GCs; 91% markedly improved, with 75% achieving complete remission of systemic disease. However, 50% of patients attaining remission later relapsed. Almost all patients suffered serious sequelae from irreversible features of their disease. Adverse effects of treatment were common: cystitis (43%), serious infection (46%), bladder cancer (2.8%), myelodysplasia (2%), GC-related cataracts (21%), fractures (11%), and osteonecrosis (3%). Sixteen women developed ovarian failure. The 2.4-fold overall increase in malignancies included a 33-fold increase in bladder neoplasms and 11-fold increase in lymphomas compared with National Cancer Institute registry contemporaneous data. The mortality was 20%.

In 1990, Hoffman *et al.* published their experience using GCs with IV pulse CYC (1 g/m^2^) monthly for six doses, modeling recent successful protocols for treatment of lupus nephritis, as an approach with similar therapeutic efficacy but less cumulative toxicity.^35,36^ Thirteen of 14 patients dramatically improved, of whom seven achieved remission within 4 months. However, nine patients later relapsed. Thus, although safer than oral CYC, the IV regimen failed to demonstrate extended efficacy.^34^ The CYCLOPS trial compared IV versus oral CYC (Table 2).^37^ One hundred forty-nine patients with newly diagnosed AAV and kidney involvement were randomized to MP and either pulse CYC or daily oral CYC. Dosage was reduced in patients age older than 70 years or for leukopenia. All patients received maintenance azathioprine (AZA) after completion of induction until 18 months after receiving the first CYC dose. The proportion of patients achieving remission by 9 months was similar in both groups (88.1% versus 87.7%, respectively) as was the time to remission. Patients in the pulse group more often relapsed, received a lower cumulative CYC dose (8.2 versus 15.9 g), and had fewer leukopenia episodes.^37,38^ This experience with CYC demonstrated not only efficacy but also significant toxicity of prolonged therapy in AAV and paved the way for multiple trials evaluating safer, more tolerable, induction, and maintenance agents.

**Table 2:** Major clinical trials for induction therapy in ANCA-associated vasculitis in chronological order

Trial Name (Year^a^)	Key Inclusion/Exclusion Criteria	Intervention	Primary Outcome	Major Highlights of Trial
MEPEX (2007) *N*=137	Inclusion: newly diagnosed GPA/MPA with kidney involvement and SCr ≥5.7 mg/dl Exclusion: life-threatening extra-renal manifestations	Both groups: oral GC; oral CYC 2.5 mg/kg per day (2 mg/kg per day for age >60 yr), reduced to 1.5 mg/kg per day after 3 mo, stopped at month 6; AZA (2 mg/kg per day) started at month 6 (up to month 12) PLEX group: seven sessions (in 2 wk, 60 ml/kg) IV MP group: 3×1000 mg	Dialysis independent at 3 mo	1. Included patients with severely reduced kidney function 2. PLEX superior for dialysis independence at 3 mo (69% versus 49%) and kidney survival at 12 mo (80% versus 57%) 3. Availability of kidney biopsy histopathological data
CYCLOPS (2009) *N*=149	Inclusion: newly diagnosed GPA/MPA with kidney involvement (SCr >1.7 mg/dl but <5.7 mg/dl)	Both groups: oral GC, AZA (2 mg/kg per day) maintenance (3 mo after remission until month 18) IV^b^: CYC three pulses 15 mg/kg (2 wk apart), then pulses every 3 wk until remission, and then for 3 mo Oral^c^: CYC 2 mg/kg per day until remission, then 1.5 mg/kg per day for 3 mo	Time to remission	1. High remission frequency in either arm at 9 mo (88%) 2. IV CYC equivalent efficacy of oral CYC with lower cumulative dose (8.2 versus 15.8 g)
RAVE (2010) *N*=197	Inclusion: newly diagnosed or relapsing GPA/MPA; SCr ≤4.0 mg/dl Exclusion: mechanical ventilation because of alveolar hemorrhage	Both groups: IV MP 1000 mg (1–3 doses), followed by oral GC RTX: 4×375 mg/m^2^ (weekly) Oral CYC: 2 mg/kg per day for 3–6 mo (adjusted for kidney function), then AZA (2 mg/kg per day) until month 18	Complete remission defined by BVAS=0 and completion of GC taper by 6 mo	1. First large RCT in AAV 2. RTX noninferior to CYC/AZA at 6 and 18 mo (64% versus 53%; 39% versus 33%) 3. RTX superior in relapsing PR3-AAV
RITUXVAS (2010) *N*=44	Inclusion: newly diagnosed GPA/MPA with kidney involvement	Both groups: IV MP 1×1000 mg, followed by oral GC RTX: 4×375 mg/m^2^ (weekly)+IV CYC (15 mg/kg) given with first and third RTX dose CYC: Pulses (15 mg/kg) at week 0, week 2, week 4, then every 3 wk for seven more doses, and then AZA (2 mg/kg per day) until month 12	Sustained remission at 12 mo and SAEs	1. Included patients with severe kidney injury 2. Equivalent frequencies of sustained remission (76% versus 82%) and frequencies of SAEs (42% versus 36%)
MYCYC (2019) *N*=140	Inclusion: newly diagnosed GPA/MPA Exclusion: life-threatening vasculitis, rapidly worsening kidney function and eGFR <15 ml/min per 1.73 m^2^	Both groups: oral GC Oral MMF: 2 g/d (increase to 3 g/d for uncontrolled AAV at week 4) for 3–6 mo IV^b^ CYC: three pulses 15 mg/kg (2 wk apart), then one pulse every 3 wk until remission, and then for 3 mo Maintenance: AZA (2 mg/kg per day) until month 18	Remission at 6 mo	1. MMF noninferior to CYC for remission induction (67% versus 61%); MMF is potential induction option when CYC or RTX contraindicated 2. Higher relapse frequency with MMF, particularly in patients with PR3-ANCA
PEXIVAS (2020) *N*=704	Inclusion: newly diagnosed GPA/MPA with kidney involvement and eGFR <50 ml/min per 1.73 m^2^ Exclusion: A comorbidity that, in the opinion of the investigator, absolutely mandates the use of PLEX	Both groups: IV MP followed by oral GC; remission induction with CYC (oral/IV, appropriate dose adjustments) or RTX (375 mg/m^2^ weekly×4) and remission maintenance for CYC users only with AZA (2 mg/kg per day) until at least week 52 2×2 factorial design 1: PLEX versus no PLEX (7 sessions within 14 d, 60 ml/kg) 2: GC standard dose versus reduced dose	Composite outcome of from any-cause death or kidney failure	1. Largest RCT to date in AAV 2. Reduced-dose GC is safe 3. Outcomes A) PLEX not superior compared with no PLEX (28.4% versus 31.0%) B) Reduced-dose GC noninferior (27.9% versus 25.5%) with fewer serious infections at 12 mo (IRR, 0.69)
ADVOCATE (2021) *N*=331	Inclusion: newly diagnosed or relapsing GPA/MPA, eGFR ≥15 ml/min per 1.73 m^2^ Exclusion: mechanically ventilated patients	Both groups: oral GC; remission induction with either CYC (oral/IV, appropriate dose adjustments) or RTX (375 mg/m^2^ weekly×4); remission maintenance for only CYC-treated with AZA (1 → 2 mg/kg per day allowed) until week 52 Intervention 1. Prednisone (60 mg/d, with tapering over 20 wk) 2. Avacopan (30 mg twice daily for 52 wk)	Remission at week 26 and sustained remission at week 52	1. Large RCT in AAV 2. First trial to test GC-free induction in AAV 3. Main outcomes A. Avacopan noninferior to GC for remission induction at week 26 (72.3% versus 70.1%) and superior to GC for sustained remission at week 52 (65.7% versus 54.9%) B. Frequencies of SAEs equivalent (37.3% versus 39%)

AAV, ANCA-associated vasculitis; AZA, azathioprine; BVAS, Birmingham Vasculitis Activity Score; CYC, cyclophosphamide; GC, glucocorticoids; GPA, granulomatosis with polyangiitis; IRR, incidence rate ratio; IV, intravenous; MMF, mycophenolate mofetil; MP, methylprednisolone; MPA, microscopic polyangiitis; PEXIVAS, Plasma Exchange and Glucocorticoids in Severe ANCA-Associated Vasculitis study; PLEX, plasma exchange; PR3, proteinase 3; RCT, randomized controlled trial; RTX, rituximab; SAE, serious adverse event; SCr, serum creatinine concentration.

a Year of publication of report.

b For intravenous cyclophosphamide, if age 60–70 years, pulse dose reduced by 2.5 mg/kg or if age older than 70 years, by 5 mg/kg; for serum creatinine >3.4 mg/dl but <5.7 mg/dl, pulse dose reduced by 2.5 mg/kg; for leukopenia, pulse dose reduced by 20% if nadir leukocyte count 2–3×10^9^/L or by 40% if nadir leukocyte count 1–2×10^9^/L.

c For oral cyclophosphamide, if age 60–70 years, pulse dose reduced by 25% or if age older than 70 years, by 50%; for leukopenia, cyclophosphamide withheld if leukocyte count <4×10^9^/L and resumed at dose reduced by 25 mg/d after resolution.

## Rise of B-Cell Therapies

The quest for an alternative induction agent for AAV with comparable/higher efficacy but less toxicity and progress in understanding the pathogenesis of AAV led to increased interest in B-cell therapies.^39^ Rituximab (RTX), a chimeric (mouse-human) anti-CD20 monoclonal antibody, showed promise as a remission-induction agent in AAV in uncontrolled studies.^40–42^ In 2010, two major trials using RTX for induction of remission in AAV were published.^43,44^ The RAVE trial compared RTX versus oral CYC (Table 2).^43^ Eligible patients with GPA or MPA had ANCA-positive serology and severe disease^10^ with Birmingham Vasculitis Activity Score (BVAS) for GPA ≥3 (scores range from 0 to 63; higher scores indicate worse disease activity).^45^ Of 197 participants with new or relapsing AAV, 102 had kidney involvement. Patients with severe kidney involvement (serum creatinine >4 mg/dl or dialysis-dependent) or severe alveolar hemorrhage were excluded. RTX was given as four weekly IV 375 mg/m^2^ doses without maintenance therapy; the comparison group received oral CYC for induction and then AZA for maintenance. RTX was noninferior in achieving the primary end point of complete remission. RTX was more efficacious as treatment for relapsing disease; 67% of patients reached the primary end point versus 42% with oral CYC.^43^ For patients with kidney involvement, improvement of eGFR and frequency of remission in the two groups were similar.^46,47^ Frequency of adverse events differed only as less pneumonia and less leukopenia with RTX.^47^ For long-term, the biological effect of RTX is variable and has been associated with hypogammaglobulinemia in AAV.^48^ Because of cost, the use of RTX remains restricted in some countries.^49^

The open-label, two-group, parallel design RITUXVAS trial sought to ascertain whether a RTX-based regimen is more effective and safer than a CYC-based regimen in patients with newly diagnosed AAV with ANCA-positive serology and kidney involvement.^44^ The intervention arm received RTX (four weekly doses) and two doses IV CYC. Controls received three doses IV CYC every 2 weeks and then every 3 weeks until stable remission and switched to oral AZA maintenance. Frequency of sustained remission, SAEs, infection, or relapse did not differ at 12 months; eGFR improved similarly in both groups.^44^ In 2011, the US Food and Drug Administration approved RTX (it is the first approved drug for AAV) when combined with GC for treatment of GPA and MPA.

## Role of Mycophenolate Mofetil

Mycophenolate mofetil inhibits *de novo* purine nucleotide synthesis, leading to decreased B-cell and T-cell proliferation and autoantibody production. It also induces apoptosis of activated T lymphocytes and inhibits lymphocyte recruitment.^50^ Numerous reports suggested a benefit of mycophenolate mofetil for induction in AAV.^51–53^ In the MYCYC study, patients with newly diagnosed AAV were randomized to receive oral mycophenolate or IV CYC and then oral AZA for remission maintenance.^51^ Patients with life-threatening vasculitis, eGFR <15 ml/min per m^2^, or RPGN were excluded. Although mycophenolate was noninferior to CYC to induce remission, there were more relapses with equivalent risk for serious infection.^51^ In a separate randomized controlled trial with 84 patients with relapsing AAV, frequency of sustained remission with mycophenolate or CYC did not differ.^52^ Mycophenolate may be considered as an alternative to CYC only in non–life-threatening vasculitis.

## Plasma Exchange as Adjunctive Induction Agent in Severe AAV

Use of plasma exchange (PLEX) in AAV was based on its benefit for antiglomerular basement membrane antibody disease.^54^ The first randomized clinical trial with PLEX for GPA, MPA, and idiopathic RPGN included 48 patients treated with oral GCs, CYC, and AZA with or without PLEX.^55^ Addition of PLEX benefitted only patients who were initially dialysis dependent. With long-term follow-up, improved kidney function was generally maintained.^55^

In 2007, the MEPEX trial tested the benefit of PLEX versus IV MP in patients with AAV with severe kidney involvement. All patients received oral CYC and oral MP with oral AZA maintenance at 6 months.^56^ Patients with severe lung hemorrhage requiring mechanical ventilation were excluded. Compared with IV MP, PLEX reduced the frequency of kidney failure at 12 months. However, survival and SAEs did not differ at 1 year; most deaths resulted from infection and lung hemorrhage.^56^ After 4 years, the short-term benefit for preservation of kidney function was lost.^57^

The recent Plasma Exchange and Glucocorticoids in Severe ANCA-Associated Vasculitis study (PEXIVAS) study enrolled 704 patients with severe AAV and randomized patients to undergo PLEX or no PLEX, in addition to induction with oral/IV CYC or RTX.^58^ The primary composite outcome was any-cause death or kidney failure. Of 660 patients randomized, 205 had severe kidney involvement and 61 had severe diffuse alveolar hemorrhage. After 7 years, frequency of death or kidney failure did not differ by treatment. There was no benefit of PLEX to induce or maintain remission, even for patients with very severe AAV.^58^

The disparate findings of the MEPEX and PEXIVAS studies may reflect differences in study eligibility, end points, and duration of follow-up. PEXIVAS was not powered to detect differences in frequency of kidney failure as an outcome independent of mortality. A meta-analysis of randomized trials, including PEXIVAS, concluded that adding PLEX to standard induction therapy did not improve mortality and may increase risk of serious infection but appeared to reduce the risk of kidney failure at 12 months.^59^ The American Society of Apheresis 2020 guidelines support PLEX to treat patients with AAV with severe AKI and pulmonary hemorrhage.^60^

## Reduced-Dose GC—Dawn of a New Era

The PEXIVAS trial also compared standard dose with reduced-dose oral GC regimens in severe AAV (Table 3). The reduced-dose group had 60% less exposure to GC at 6 months and was noninferior to standard dose for all-cause mortality and kidney failure at 12 months. Post hoc analysis showed neither difference in efficacy between the two GC regimens for patients with severe kidney disease or alveolar hemorrhage, although reduced-dose patients had fewer severe infections, nor between the RTX versus CYC regimens.^58^ With contemporaneous treatment regimens improving survival of patients with AAV, infections rather than active vasculitis became the major cause of mortality in the first treatment year,^61^ and GC therapy was a risk factor.^62,63^ The 2021 Kidney Disease Improving Global Outcomes guidelines^64^ and the 2022 European Alliance of Associations for Rheumatology guidelines recommend reduced-dose GC therapy for the treatment of AAV.^65^

**Table 3:** Glucocorticoid dosing in plasma exchange and glucocorticoids in severe ANCA-associated vasculitis study

Week	Standard-Dose Arm			Reduced-Dose Arm		
	<50 kg	50–75 kg	>75 kg	<50 kg	50–75 kg	>75 kg
	Pulse	Pulse	Pulse	Pulse	Pulse	Pulse
1	50	60	75	50	60	75
2	50	60	75	25	30	40
3–4	40	50	60	20	25	30
5–6	30	40	50	15	20	25
7–8	25	30	40	12.5	15	20
9–10	20	25	30	10	12.5	15
11–12	15	20	25	7.5	10	12.5
13–14	12.5	15	20	6	7.5	10
15–16	10	10	15	5	5	7.5
17–18	10	10	15	5	5	7.5
19–20	7.5	7.5	10	5	5	5
21–22	7.5	7.5	7.5	5	5	5
23–52	5	5	5	5	5	5
>52	Investigators' local practice					

Glucocorticoid dosing in mg/d.

## Drugs Targeted against Complement System—New Kids on the Block

Complement-based therapies have been developed as GC-sparing regimens for treatment of AAV. Avacopan, an oral selective-C5a-receptor inhibitor, inhibits neutrophilic chemoattraction and activation. In the phase II CLEAR trial,^66^ avacopan was noninferior to oral GCs for >50% decrease in BVAS with a trend for faster remission.^66^ Avacopan was further investigated in the phase III ADVOCATE trial^67^ with 331 patients with new or relapsing ANCA-positive AAV and eGFR ≥15 ml/min per m^2^ receiving oral avacopan 30 mg twice daily or oral prednisone on a tapering schedule. All patients had induction therapy with oral/IV CYC (AZA for maintenance) or RTX. The first primary end point of remission at week 26 (BVAS=0) with no GC use in the preceding 4 weeks was similar in both groups. Sustained remission through 52 weeks was more common with avacopan. SAEs were similar between groups.^67^ Infection, especially with encapsulated organisms, was uncommon, likely because avacopan does not block formation of C5b and the membrane attack complex.^67^ There are few data about use of avacopan beyond 1 year, optimal timing of discontinuation, and effect of C5-mediated systemic injury after stopping avacopan. The US Food and Drug Administration and European Medicines Agency have approved avacopan for AAV combined with standard induction therapy.

Eculizumab, a recombinant humanized monoclonal antibody targeting complement protein C5 to block formation of the complement membrane attack complex, has been effective in some refractory cases with aggressive AAV-induced end-organ damage.^68–70^ Vilobelimab, a human-mouse chimeric IgG4 kappa monoclonal antibody, binds to human C5a. Preliminary results of the phase II IXCHANGE trial showed that vilobelimab more often than GC induced remission in patients with GPA or MPA who received CYC-based or RTX-based induction, with substantial reduction in GC dose and toxicity.^71^

## Evolution of Maintenance Therapy in AAV

Patients with AAV attaining remission after induction therapy usually start maintenance therapy to decrease risk of relapse. Patients with proteinase 3 (PR3)-ANCA and upper respiratory tract or lung involvement have a higher risk for relapse.^72,73^ To date, the optimal duration of maintenance therapy remains unknown. To reduce exposure to cytotoxic drugs, AZA was compared with oral CYC in the 2003 CYCAZAREM trial; patients with newly diagnosed AAV were randomized to oral CYC or AZA for 12 months for maintenance after remission induced with oral CYC and IV GCs (Table 4).^74^ Frequency of relapse and adverse events were similar in the groups after 18 months. In the 2008 WEGENT trial, AZA was compared with oral methotrexate for remission maintenance at 12 months after induction with IV CYC and GCs; similar frequency of relapse and adverse effects persisted long term.^75,76^ The IMPROVE study showed that AZA was superior to mycophenolate for maintenance after induction with oral/IV CYC.^77^

**Table 4:** Major clinical trials for maintenance therapy in ANCA-associated vasculitis in chronological order

Trial Name (Year^a^)	Key Inclusion/Exclusion Criteria	Intervention	Primary Outcome	Major Highlights of Trial
CYCAZAREM (2003) *N*=155	Inclusion: newly diagnosed GPA/MPA, SCr <5.7 mg/dl, attained remission with oral CYC and GC for at least 3 mo (*n*=144) Exclusion: cytotoxic drug use in prior 12 mo, SCr ≥5.7 mg/dl	144 patients entered remission Oral CYC (continued at 1.5 mg/kg per day) versus AZA (2 mg/kg per day) for total of 12 mo	Relapse at month 18	1. Long-term CYC had significant side effects 2. AZA suitable as a replacement for CYC 3. Relapse frequency similar at 18 mo (10% [CYC] versus 11% [AZA]) 4. Similar frequency of SAEs 5. First study to show efficacy of short-course oral CYC
WEGENT (2008) *N*=126	Inclusion: newly diagnosed GPA/MPA with remission after pulse CYC and GC	AZA (2 mg/kg per day) versus oral MTX (0.3 mg/kg per week with progressive increase to 25 mg/wk) for total of 12 mo	Adverse event causing discontinuation/death at 29 mo	1. Adverse events and SAEs similar between AZA and MTX (29/63 versus 35/63, *P* = 0.29; 5/63 versus 11/63, *P*=0.11, respectively) 2. Primary end point similar between AZA and MTX (7/63 versus 12/63, *P* = 0.21) 3. Relapse frequency on AZA or MTX similar (23/63 versus 21/63, *P* = 0.71) 4. Most relapses occurred after AZA or MTX stopped (32/44)
IMPROVE (2010) *N*=156	Inclusion: newly diagnosed GPA/MPA and attained remission with oral CYC and GC Exclusion: cytotoxic drug use in prior 12 mo, failure to achieve remission after 6 mo CYC, or progressive disease	AZA: 2 mg/kg per day; reduced to 1.5 mg/kg per day after 12 mo, then to 1 mg/kg per day 18 mo; withdrawal at 42 mo MMF: 2000 mg/d; reduced to 1500 mg/d after 12 mo and 1000 mg/d after 18 mo; withdrawal at month 42	Relapse-free survival	1. AZA superior to MMF 2. Higher relapse frequency with MMF (42/76 MMF versus 30/80 AZA)
MAINRITSAN (2014) *N*=115	Inclusion: newly diagnosed or relapsing GPA/MPA with remission after pulse CYC and GC	RTX: 2×500 mg IV (14 d apart), then 500 mg IV at month 6, month 12, and month 18 (total 18 mo) AZA: 2 mg/kg per day for 12 mo, then 1.5 mg/kg per day for 6 mo, and then 1 mg/kg per day for 4 mo (total 22 mo)	Major relapse at month 28	1. RTX superior to AZA in remission maintenance: major relapses, 5% versus 29%; minor relapses, 11% versus 16% 2. SAEs similar (19% versus 14%), particularly in patients with PR3-ANCA patients
MAINRITSAN 2 (2018) *N*=162	Inclusion: newly diagnosed or relapsing GPA/MPA with remission after IV/oral CYC and GC, IV/oral MTX and GC, or RTX and GC	Fixed-dose RTX: 500 mg IV on day 0 and day 14 and at month 6, month 12, and month 18 (total 18 mo) Tailored-dose RTX: 500 mg IV at randomization; peripheral B cells and ANCA serologies measured every 3 mo, further RTX administered if CD19 count >0, ANCA-positive serology reappeared, or ANCA titer doubled from baseline (total 18 mo)	Major relapse at month 28	1. Large trial comparing two RTX regimens; tailored dose noninferior to fixed dose 2. Relapse in 14/81 patients (major relapse=6) with tailored dose versus 8/81 (major relapse=3) with fixed dose (17.3% versus 9.9%) 3. Fewer SAEs with tailored dose versus fixed dose (37/81 versus 53/81) 4. Fewer infusions and lower cumulative dose with tailored dose
BREVAS (2019) *N*=105	Inclusion: newly diagnosed or relapsing GPA/MPA with remission after IV/oral CYC and GC or RTX and GC	AZA (2 mg/kg per day)+oral prednisone (≤10 mg/d)+either 1. IV belimumab 10 mg IV on day 0, day 14, day 28, and then every 28 d until study completion or relapse 2. IV placebo on day 0, day 14, day 28, and then every 28 d until study completion or relapse	I. Time to first PSE 1. BVAS ≥6 2. ≥1 major BVAS item 3. Additional IS use 4. Treatment failure II. Vasculitis relapse	1. Belimumab did not reduce risk of PSE compared with placebo (HR, 1.07; 95% CI, 0.44 to 2.59; *P* = 0.884) 2. Belimumab did not reduce vasculitis relapse compared with placebo (HR, 0.88; 95% CI, 0.29 to 2.65; *P* = 0.821) 3. All vasculitis relapses in belimumab group (*N*=6) occurred in PR3-ANCA patients with CYC induction 4. Vasculitis relapse in the placebo group (*N*=8) occurred independent of induction agent, disease stage, or ANCA type 5. Overall frequency of PSEs low (18.9% in belimumab group versus 21.2% in placebo group)
MAINRITSAN 3 (2020) *N*=97	Inclusion: patients with GPA/MPA in sustained remission after completing MAINRITSAN 2	Randomization at month 28 1. RTX arm: 500 mg at month 0, month 6, month 12, and month 18 (total 18 mo) 2. Placebo infusion at month 0, month 6, month 12, and month 18 (total 18 mo)	Relapse-free survival at month 28	1. RTX given after long-term remission (28 mo) superior to no further maintenance therapy 2. Relapse-free survival (RTX versus placebo): 96% versus 74%, major relapse-free survival (RTX versus placebo): 100% versus 87% 3. SAEs (RTX versus placebo): 24% versus 30%
RITAZAREM (2023) *N*=188	Inclusion: relapsed GPA/MPA after re-induction with RTX and GC Exclusion: B-cell depleting agent in prior 6 mo or biologic in prior 3 mo	RTX: 1000 mg IV every 4 mo×5 doses (total 20 mo) AZA: 2 mg/kg per day, tapered after month 24 (total up to 27 mo)	Time to relapse	1. RTX superior to AZA in patients with relapsing disease (HR, 0.41, *P* < 0.0001) 2. Fewer SAEs with RTX (19/85 [22%] versus 31/85 [36%]) 3. Frequency of hypogammaglobulinemia did not differ by treatment

AZA, azathioprine; BVAS, Birmingham Vasculitis Activity Score; CI, confidence interval; CYC, cyclophosphamide; GC, glucocorticoids; GPA, granulomatosis with polyangiitis; HR, hazard ratio; IS, immunosuppressant; IV, intravenous; MMF, mycophenolate mofetil; MPA, microscopic polyangiitis; MTX, methotrexate; PR3, proteinase 3; PSE, protocol-specified event; RTX, rituximab; SAE, serious adverse event; SCr, serum creatinine concentration.

a Year of publication of report.

Over the past decade, RTX has been increasingly used for maintenance of remission in AAV. The MAINRITSAN trial randomized 115 patients, most with new-onset AAV, who had received IV CYC for induction, to oral AZA or RTX.^78^ RTX reduced frequency of relapse with a comparable severe adverse effect profile. In the RITAZAREM trial, RTX was better than AZA as maintenance therapy in 188 patients with relapsing AAV after induction with RTX.^79^

Optimal dose and duration of RTX as maintenance therapy were investigated in two other trials. MAINRITSAN-2 randomized patients with newly diagnosed or relapsing GPA or MPA in complete remission after induction to receive fixed-dose (500 mg every 6 months) or a tailored-dose regimen (500 mg on >2-fold increase in ANCA titers, ANCA negative-to-positive seroconversion, or peripheral B-cell restoration). RTX was administered up to 18 months. At 28 months, frequency of relapse and SAEs did not differ. However, patients in the tailored arm received fewer infusions with lesser cumulative dose.^80^ Participants who completed follow-up in MAINRITSAN-2 and were still in remission were randomized in MAINRITSAN-3 to RTX or placebo. Placebo patients, mostly with PR3-AAV, relapsed more often.^81^ Thus, low-dose RTX maintains remission and long-term treatment lessens relapse, particularly in patients with PR3-AAV.

With beneficial outcomes for patients with AAV receiving RTX, alternative therapies targeting B cells have been tested. Belimumab, a human monoclonal antibody against B-cell activating factor, was evaluated in the BREVAS trial in patients with AAV in remission after induction with oral/IV CYC or RTX. IV belimumab plus AZA was not more efficacious than AZA alone for reducing relapse, perhaps because of unexpectedly few relapses in the latter group.^82^

In conclusion, AAV is a rare multisystem disease identified almost a century ago that frequently damages kidneys. Before use of immunosuppressants, severe AAV was almost universally fatal. Over the past four decades, great strides in understanding the disease immunopathogenesis have led to effective, safer, targeted therapies to induce remission and reduce complications. This evolution of therapy for AAV has markedly improved the outlook for patients.
